# Noisy Interlimb Coordination Can Be a Main Cause of Freezing of Gait in Patients with Little to No Parkinsonism

**DOI:** 10.1371/journal.pone.0084423

**Published:** 2013-12-31

**Authors:** Takao Tanahashi, Tomohisa Yamamoto, Takuyuki Endo, Harutoshi Fujimura, Masaru Yokoe, Hideki Mochizuki, Taishin Nomura, Saburo Sakoda

**Affiliations:** 1 Department of Neurology, Osaka University Graduate School of Medicine, Osaka, Japan; 2 Osaka University Graduate School of Engineering Science, Osaka, Japan; 3 Department of Neurology, Toneyama National Hospital, Osaka, Japan; Centre national de la recherche scientifique, France

## Abstract

Freezing of gait in patients with Parkinson’s disease is associated with several factors, including interlimb incoordination and impaired gait cycle regulation. Gait analysis in patients with Parkinson’s disease is confounded by parkinsonian symptoms such as rigidity. To understand the mechanisms underlying freezing of gait, we compared gait patterns during straight walking between 9 patients with freezing of gait but little to no parkinsonism (freezing patients) and 11 patients with Parkinson’s disease (non-freezing patients). Wireless sensors were used to detect foot contact and toe-off events, and the step phase of each foot contact was calculated by defining one stride cycle of the other leg as 360°. Phase-resetting analysis was performed, whereby the relation between the step phase of one leg and the subsequent phase change in the following step of the other leg was quantified using regression analysis. A small slope of the regression line indicates a forceful correction (phase reset) at every step of the deviation of step phase from the equilibrium phase, usually at around 180°. The slope of this relation was smaller in freezing patients than in non-freezing patients, but the slope exhibited larger step-to-step variability. This indicates that freezing patients executed a forceful but noisy correction of the deviation of step phase, whereas non-freezing patients made a gradual correction of the deviation. Moreover, freezing patients tended to show more variable step phase and stride time than non-freezing patients. Dynamics of a model of two coupled oscillators interacting through a phase resetting mechanism were examined, and indicated that the deterioration of phase reset by noise provoked variability in step phase and stride time. That is, interlimb coordination can affect regulation of the gait cycle. These results suggest that noisy interlimb coordination, which probably caused forceful corrections of step phase deviation, can be a cause of freezing of gait.

## Introduction

Freezing of gait (FOG) occurs when patients are temporarily unable to generate effective stepping, usually for only a few seconds, but can walk relatively smoothly after FOG is overcome [1], [2]. Parkinson’s disease (PD) is a representative cause of FOG [3], but more than half of patients with atypical parkinsonism such as progressive supranuclear palsy and multiple system atrophy also experience FOG [4], [5], and patients with pure akinesia [6], [7] or primary progressive freezing gait [8], [9] experience FOG with no or little parkinsonism early in the course of disease. The underlying mechanisms of FOG are not fully understood [10], [11].

Poor coordination between the legs is associated with FOG. Several studies have reported that the relative step phase between the legs during straight walking was more variable and further from 180° in PD patients with FOG (PD+FOG) than in PD patients without FOG (PD−FOG) [12], [13], [14]. A study of left-right independent pedaling movements reported that the relative phase between the legs deviated from 180° but was stable in PD−FOG, but varied quasi-periodically or irregularly in PD+FOG [15], suggesting that step phase variability was more closely associated with FOG than the deviation of step phase from 180°. FOG occurs frequently during turning, which requires a greater degree of interlimb coordination than straight walking [16]. These results indicate that there is a relation between interlimb coordination and FOG.

Impaired regulation of the gait cycle is also associated with FOG. Cadence increased during FOG episodes [17] and stride time variability during straight walking was higher in PD+FOG than in PD−FOG [18]. In addition, cadence was higher during turning than during straight walking in PD+FOG but not in PD−FOG or healthy subjects [19], [20]. These findings suggest that increased cadence during turning causes gait festination and leads to FOG. The relation between regulation of the gait cycle and interlimb coordination is still unknown, but impaired regulation of the gait cycle can be aggravated by poor interlimb coordination.

The majority of previous studies have analyzed gait abnormalities in PD+FOG during off medication. However, gait analysis in PD patients is confounded by rigidity and ‘wearing off’ phenomena such as dyskinesias that directly affect gait. In addition, although the deviation of step phase from 180° has been reported to be associated with FOG [12], [13], [14], this may be affected by the asymmetry that can be particularly high in PD [12]. These confounding factors can be avoided by studying gait patterns in patients with FOG but little to no parkinsonism.

We hypothesized that step phase variability would be more closely associated with FOG than the deviation of step phase from 180°, and that step phase variability would affect stride time variability, leading to gait festination and FOG. We considered that interlimb coordination might be determined primarily by neural interaction between the left and right neural centers that regulate gait, such as reciprocal inhibition and mutual excitation, therefore we quantified the strength and the stochastic variability of the neural interaction between the two centers, and constructed a mathematical model of two oscillators coupled by a phase resetting mechanism to shed light on the mechanisms of interlimb coordination.

## Patients and Methods

### Subjects

Patients were recruited from the FOG clinic at Toneyama National Hospital between September 2010 and September 2012. Patients were diagnosed as FOG with little to no parkinsonism (FOG−P) if they experienced FOG within 3 years of the onset of symptoms, had no or very mild rigidity, no neurological abnormalities other than little to no parkinsonism, and no specific abnormalities on brain magnetic resonance images. Patients were excluded from the FOG−P group if they reported FOG but showed no FOG in the examination room or during gait measurements. The FOG−P group was not comprised of patients with a single disease, but of patients with a heterogeneous syndrome including pure akinesia, primary progressive freezing gait, and PD, among others. Patients were diagnosed as PD−FOG if they were diagnosed with PD by UK Brain Bank criteria [21], had Hoehn and Yahr stage 3, and had no FOG. Patients were excluded from this study if they had complications that affected gait such as a history of stroke and orthopedic diseases.

Motor symptoms were assessed using part III of the Unified Parkinson’s Disease Rating Scale (UPDRS) [22] in the early afternoon, after regular medications had been taken. The severity of FOG was evaluated using a new FOG questionnaire [23]. Patients in the FOG−P group were examined with cardiac scintigraphy with metaiodobenzylguanidine.

This study was approved by the Institutional Review Board of Toneyama National Hospital, and was performed in accordance with the principles expressed in the Declaration of Helsinki. All subjects gave written informed consent before the gait measurements.

### Apparatus and Procedures

Small wireless hybrid sensors (WAA-006, Wireless Technologies, Inc., Tokyo, Japan) were used to synchronously measure three-axis acceleration and angular velocity. The size of each sensor was 30×44×12 mm, and the weight was 20 g. The range of the accelerometer was ±4 G, and the range of the gyro sensor was ±300°/s. These sensors were attached to the respective heels of nursing shoes worn by the patients. AccelLoggerCE for Windows Mobile (ATR-Promotions, Inc., Kyoto, Japan) was used to collect data from these sensors via Bluetooth. The sampling frequency was 100 Hz. There are several devices for gait analysis, such as foot pressure sensors (insole or floor mat), force plates, and motion capture systems. Several studies have performed gait analyses in PD patients using accelerometers or gyroscopes recently, because these sensors do not require a special laboratory, do not interfere with locomotion, and enable long-term monitoring [24], [25], [26], [27].

Patients had medical examinations in the early afternoon, after taking their regular medications. They walked 20 m in a straight line, turned 180° around a chair clockwise or counter-clockwise, and walked 20 m to return to the start at their preferred speed. They performed this two times for each turn direction. A doctor followed the patients to prevent falls. A researcher carrying a Windows Mobile also followed to prevent interruption of wireless communication, and shook an accelerometer (WAA-001, Wireless Technologies, Inc.) at the start and the end of turn. This accelerometer was synchronized with the sensors that were attached to the patient, therefore the period of turn could be identified in the recorded data. Another researcher made video recordings of the trials so that FOG episodes could be visually assessed offline.

### Data Analysis

**Figure 1:**
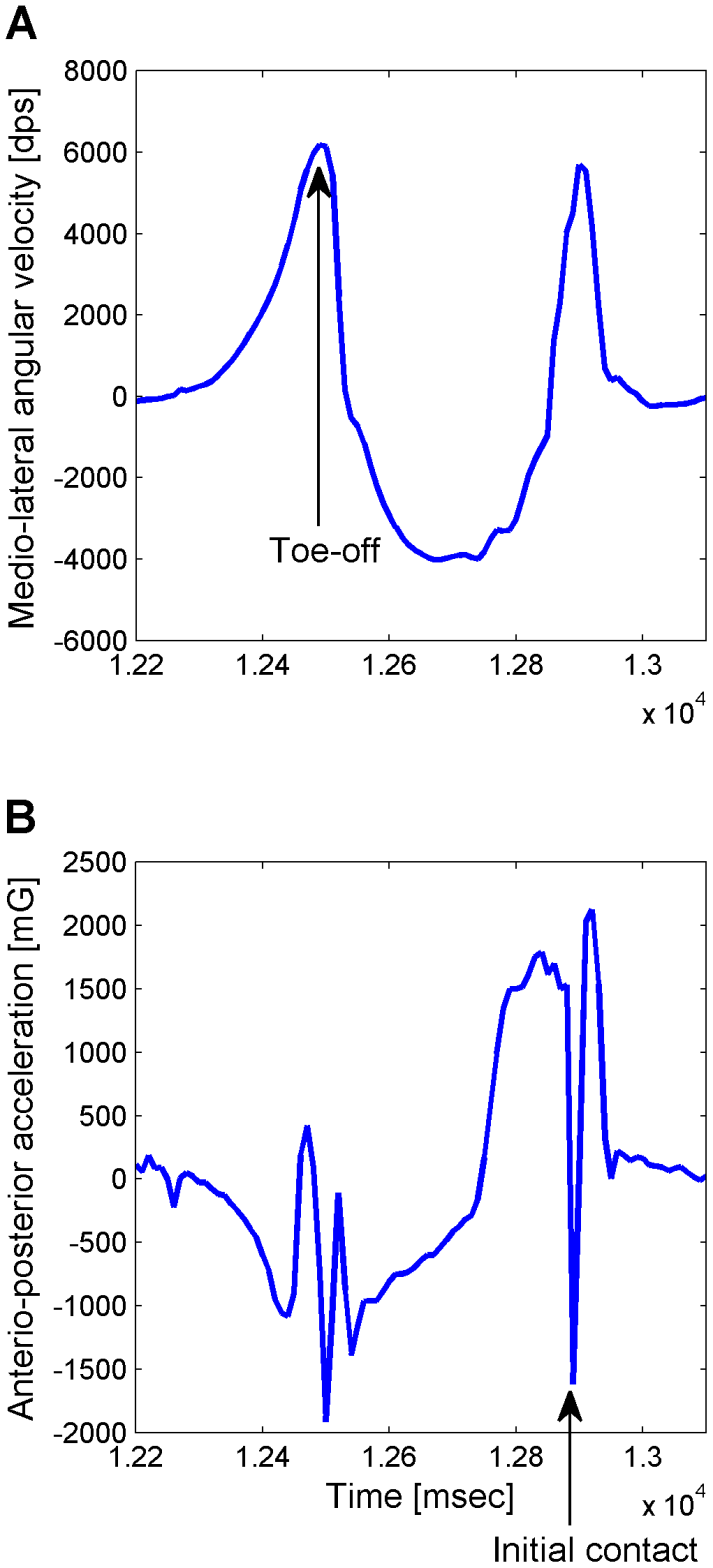
The identification of toe-off and initial foot contact events. A: Angular velocity of the heel around the medio-lateral axis. The angular velocity increased when the heel left the ground, and it decreased at the swing phase after the toe left the ground. Toe-off event was defined as the positive peak after the heel left the ground. B: Linear acceleration of the ankle in the anterio-posterior direction. The acceleration increased at the swing phase, and initial foot contact event was defined as the negative peak when the foot landed the ground after the swing phase.

MATLAB (MathWorks, Inc., Natick, Massachusetts, USA) was used for data processing. Toe-off and initial foot contact events were determined for each step (Figure 1) and used to calculate stride time, step time, swing time, and stance time. The step phase (*φ*) at which each foot contact occurred was calculated by defining the stride cycle of the other leg as 360° [12].

The straight-walking data were analyzed separately for the ‘Go’ and ‘Back’ portions of the walking task, and the first and the last 4 steps of each portion were excluded. The coefficient of variation (CV) of stride time was calculated for each leg and the mean of the two legs was used as a measure of stride time variability [18]. The CV of step phase (*φCV*) and the mean absolute deviation of step phase from 180° (*φ_deviation*) were calculated for each leg, and the mean of the two legs was used as a measure of step phase variability and step phase deviation respectively [12].
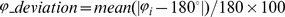



To investigate how lengthening or shortening of a given step affected the following step made by the contralateral leg, the amount of phase correction at every foot contact was estimated using the step phase of the preceding (contralateral) foot contact. Specifically, the step phase change for the (*i*+1)-th step (Δ*φ_i_*) was calculated as the difference between the step phase of the *i*-th step (*φ_i_*) and the step phase of the following (*i*+1)-th step made by the contralateral leg (*φ_i_*_+1_).
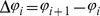



Δ*φ_i_* for each step made by the left leg was plotted against *φ_i_* (the step phase of the preceding step made by the right leg) and Δ*φ_i_* for each step made by the right leg was plotted against *φ_i_* (the step phase of the preceding step made by the left leg). The two relations were quantified using linear regression. The slope of the relation (*slope*) indicated the strength of step phase correction between the legs, and the intercept with the horizontal axis (*intercept*) indicated the equilibrium step phase (where the phase correction was zero). The mean slope of the two regression lines (*slope_mean*) was used to evaluate the strength of the step phase correction and the difference in the intercept between the two lines (*φ_asymmetry*) was used to evaluate the asymmetry in the equilibrium phase. The slope was generally negative, because Δ*φ_i_* will be positive if *φ_i_* is smaller than the equilibrium phase, and Δ*φ_i_* will be negative if *φ_i_* is larger than the equilibrium phase. If the slope is −1, Δ*φ_i_* is identical to the deviation of *φ_i_* from the equilibrium phase, and *φ_i+1_* is equal to the equilibrium phase. Therefore, the step phase is adjusted to the equilibrium phase more forcefully as the slope is closer to −1.

The two regression lines were used to predict *φ_i_*_+1_ based on the following autoregressive model:
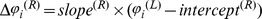


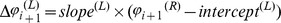


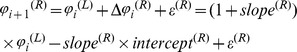


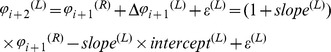
where *φ_i_* ^(L)^ and *φ_i+2_* ^(L)^ represent the step phase for the *i*-th and the (*i*+2)-th left step, respectively, and *φ_i+1_* ^(R)^ represents the step phase for the (*i*+1)-th right step. *ε* ^(R)^ and *ε* ^(L)^ represent the noise. The mean standard deviation of the noise (Δ*φ_error*) was calculated to evaluate the magnitude of noise in the step phase correction.

Δ*φ_i_* represents the same information as the phase resetting curve that has previously been used to analyze biological rhythms [28]. See Appendix S1 for the relation between Δ*φ_i_* and the phase resetting curve. We constructed a mathematical model of two coupled phase-oscillators, in which two oscillators interacted via the phase resetting curves (Appendix S1) [28], [29]. Dynamics of the model were numerically simulated to understand the step phase correction more systematically. To this end, we investigated how the dynamics of the model depended on the slope and the intercept in the regression analysis and the amount of noise in the phase reset. See Appendix S1 for details.

### Statistical Analysis

MATLAB and Microsoft Excel 2007 (Microsoft Japan Co., Tokyo, Japan) were used for statistical analysis. Gait parameters were averaged over the four trials of straight walking. If a patient showed start hesitation, the steps during FOG episodes were excluded from the analysis. If a patient showed frequent FOG during straight walking, the trial was excluded from the analysis. FOG episodes were assessed from video recordings by at least two neurologists. Data are reported as mean ± standard deviation. Equality of variance between the two groups was tested using an F-test. Clinical features were compared across FOG–P and PD–FOG groups using an unpaired t-test for variables with equal variance and a Welch’s t-test for variables with unequal variance. A Fisher’s exact probability test was used for categorical data. A two-way analysis of variance was used to investigate the effects of group (FOG–P and PD–FOG) and walking condition (‘Go’ and ‘Back’) on the gait parameters. A *P* value less than 0.05 was considered statistically significant.

## Results

### Clinical Features

Analysis was performed on 9 FOG–P and 11 PD–FOG (Table S1). Clinical features of these patients are summarized in Table 1. Age was not different between the groups (*P* = 0.18), but disease duration was shorter in FOG–P than in PD–FOG (*P*<0.05). The total UPDRS part III score was lower in FOG–P than in PD–FOG (*P*<0.01), but the axial score (total score of standing, posture, gait, and postural instability items) and the total repetitive movement score of the lower limbs were not different between the groups (*P* = 0.36 and *P* = 0.61, respectively). FOG–P showed little to no rigidity, and the total rigidity score of the neck and limbs was lower in FOG–P than in PD–FOG (*P*<0.01). Eight of the 9 FOG–P patients were examined with the cardiac scintigraphy with metaiodobenzylguanidine, and all had normal results.

**Table 1:** Patients’ clinical features.

	FOG–P	PD–FOG	*P* value
Age (years)	72.9±5.8	69.2±6.0	0.18
Gender (M/F)	2/7	7/4	0.09
Disease duration (years)	2.7±0.8	6.1±5.0	<0.05
FOG onset (years)	1.0±1.0		
UPDRS III score			
Total	18.6±6.8	31.1±11.1	<0.01
Axial	5.7±1.7	5.0±1.5	0.36
Upper limb movement	5.4±4.3	9.4±3.7	<0.05
Lower limb movement	2.8±1.5	2.4±2.0	0.61
Rigidity	1.1±0.8	7.5±4.4	<0.01
Tremor	0.3±0.7	2.5±1.8	<0.01
NFOG-Q score			
Part 2	15.8±0.9		
Part 3	6.6±1.7		

FOG–P: Patients with freezing of gait (FOG) with little to no parkinsonism.

PD–FOG: Patients with Parkinson’s disease without FOG.

FOG onset: The time from symptom onset to FOG onset.

UPDRS: Unified Parkinson’s disease rating scale.

Axial: The total of standing, posture, gait and postural instability items.

Upper limb movement: The total of upper limb repetitive movement items.

Lower limb movement: The total of lower limb repetitive movement items.

Rigidity: The total rigidity score for the neck and limbs.

Tremor: The total tremor score for the neck and limbs.

NFOG-Q: New freezing of gait questionnaire.

**Figure 2:**
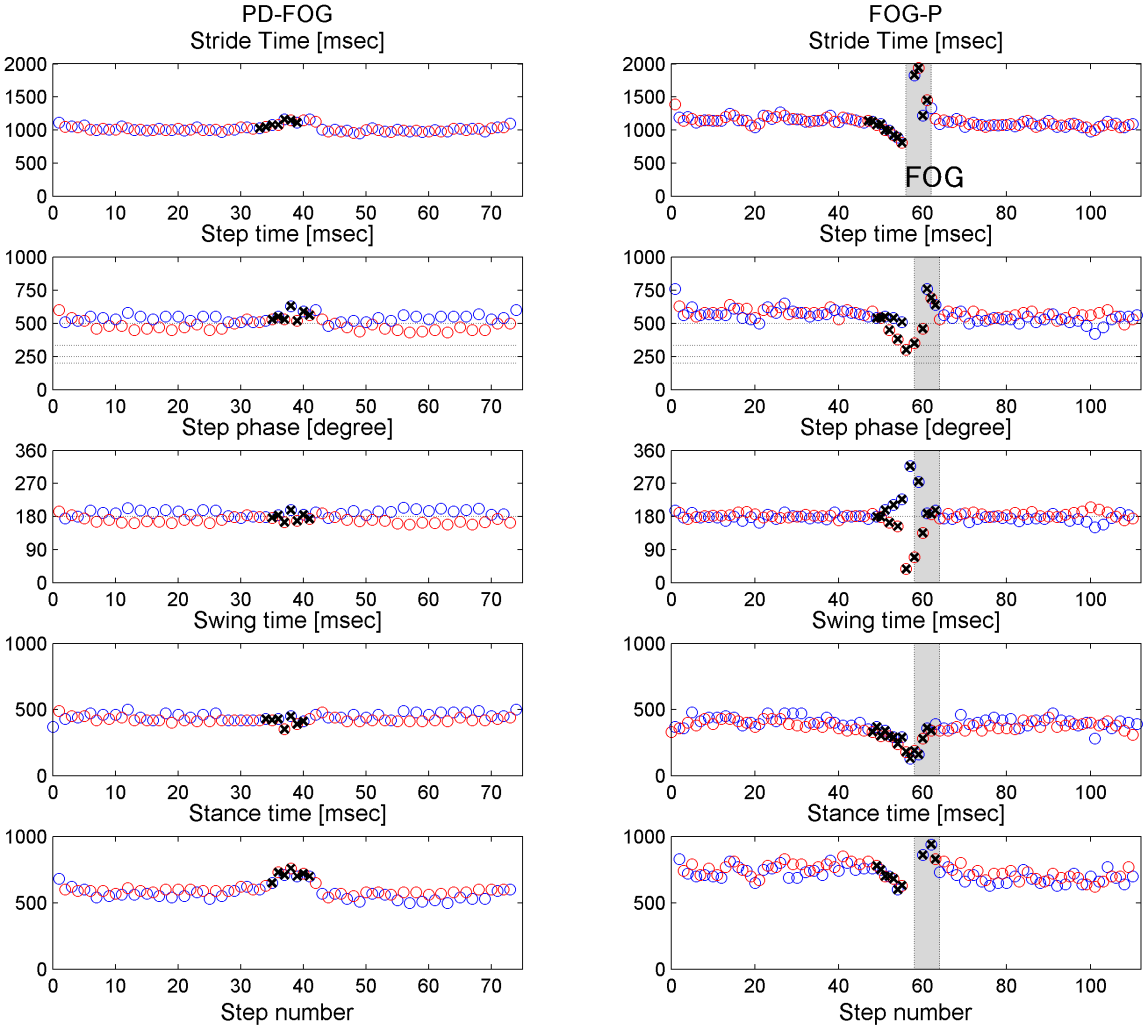
Representative sequences of gait parameters. Stride time, step time, step phase, swing time and stance time during a single walking trial (20 m straight walk, 180° clockwise turn, and 20 m straight walk) in a PD−FOG patient (left) and a FOG−P patient (right). Blue and red circles represent the data from the left and the right leg, respectively. Crosses marks indicate the data recorded during the turn. Grey shading represents FOG. The PD−FOG patient exhibited deviation of step phase from 180° but stable stride time and step phase during straight walking, and slightly increased stride time and asymmetric step phase during the turn. In contrast, the FOG−P patient exhibited large variability in stride time and step phase with step phase close to 180° during straight walking, and reduced stride time and increased step phase deviation during the turn, which preceded FOG.

All 9 FOG–P patients showed turn hesitation, and 4 FOG–P patients had incidences of start hesitation. Leg movements during all FOG episodes were shuffling with small steps or trembling in place [30]. Figure 2 shows representative sequences of gait parameters. In PD–FOG the step phase and stride time were relatively stable during straight walking, even if the step phase deviated from 180°, and there was swing time asymmetry. FOG–P tended to exhibit larger variability in stride time and step phase than PD–FOG, even if the step phase was closer to 180° than in PD–FOG. During turning, stride time, in particular stance time, increased and step phase exhibited slight asymmetry in PD–FOG patients, similar to previous reports in healthy subjects [31], [32]. In contrast, stride time decreased and the deviation of step phase from 180° was large in FOG–P patients, and FOG was common.

### Gait Parameters during Straight Walking

**Figure 3:**
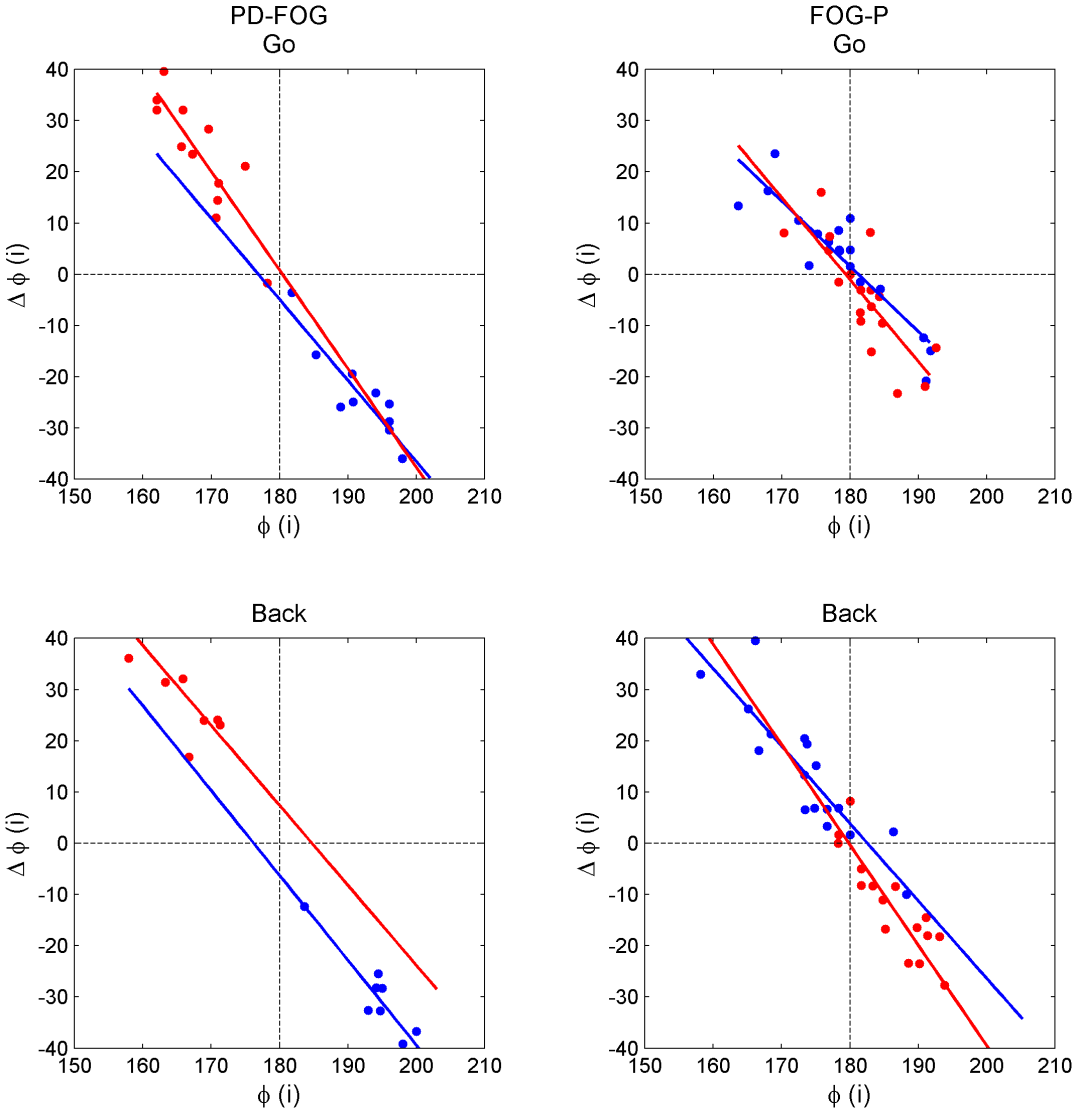
Step phase regulation. Linear regression analyses between the relative step phase of each step (*φ_i_*) and the phase change from each step to the following step (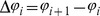) in a PD−FOG patient (left) and a FOG−P patient (right) during the ‘Go’ (upper) and ‘Back’ (lower) portions of the walking task. The analyses were performed separately for the left-to-right phase changes (blue) and for the right-to-left phase changes (red). The slope of the relation was smaller and the noise in the step phase regulation was larger in FOG−P than in PD−FOG.

*Stride time CV* and *φCV* were slightly higher in FOG–P than in PD–FOG, but these differences were not significant (*P* = 0.07 and *P* = 0.06, respectively; Table 2). *φ_deviation* was not different between the groups (*P* = 0.62; Table 2). Figure 3 shows representative results from the regression of Δ*φ_i_* and *φ_i_*. *Slope_mean* was smaller and closer to –1 and Δ*φ_error* was larger in FOG–P than in PD–FOG (*P*<0.05 and *P*<0.01, respectively; Table 2), but *φ_asymmetry* was not different between the groups (*P* = 0.30; Table 2). These results suggest that the step phase correction was more forceful but more noisy in FOG–P than in PD–FOG.

**Table 2:** Gait parameters during straight walking.

	Go		Back		P value		
	FOG–P	PD–FOG	FOG–P	PD–FOG	Group	Condition	Interaction
*Stride time CV*	3.58±1.47	2.46±1.97	4.25±2.91	2.45±0.75	0.07	0.39	0.38
*φCV*	3.21±0.97	2.44±0.80	3.44±1.16	2.56±0.95	0.06	0.29	0.73
*φ_deviation*	2.99±0.91	2.95±1.25	3.47±0.95	3.01±1.26	0.62	0.11	0.21
*Slope_mean*	−1.50±0.09	−1.57±0.10	−1.47±0.08	−1.59±0.10	0.02	0.99	0.54
*φ_asymmetry*	2.25±1.26	2.29±1.47	3.41±1.17	2.59±1.75	0.30	0.04	0.16
Δ*φ_error*	8.73±2.39	6.58±3.29	22.27±12.56	7.36±2.44	<0.01	<0.01	<0.01

FOG–P: Patients with freezing of gait (FOG) with little to no parkinsonism.

PD–FOG: Patients with Parkinson’s disease without FOG.

Group: The main effects of group (FOG–P and PD–FOG).

Condition: The main effects of walking condition (‘Go’ and ‘Back’).

Interaction: The interaction between group and walking condition.

**Figure 4:**
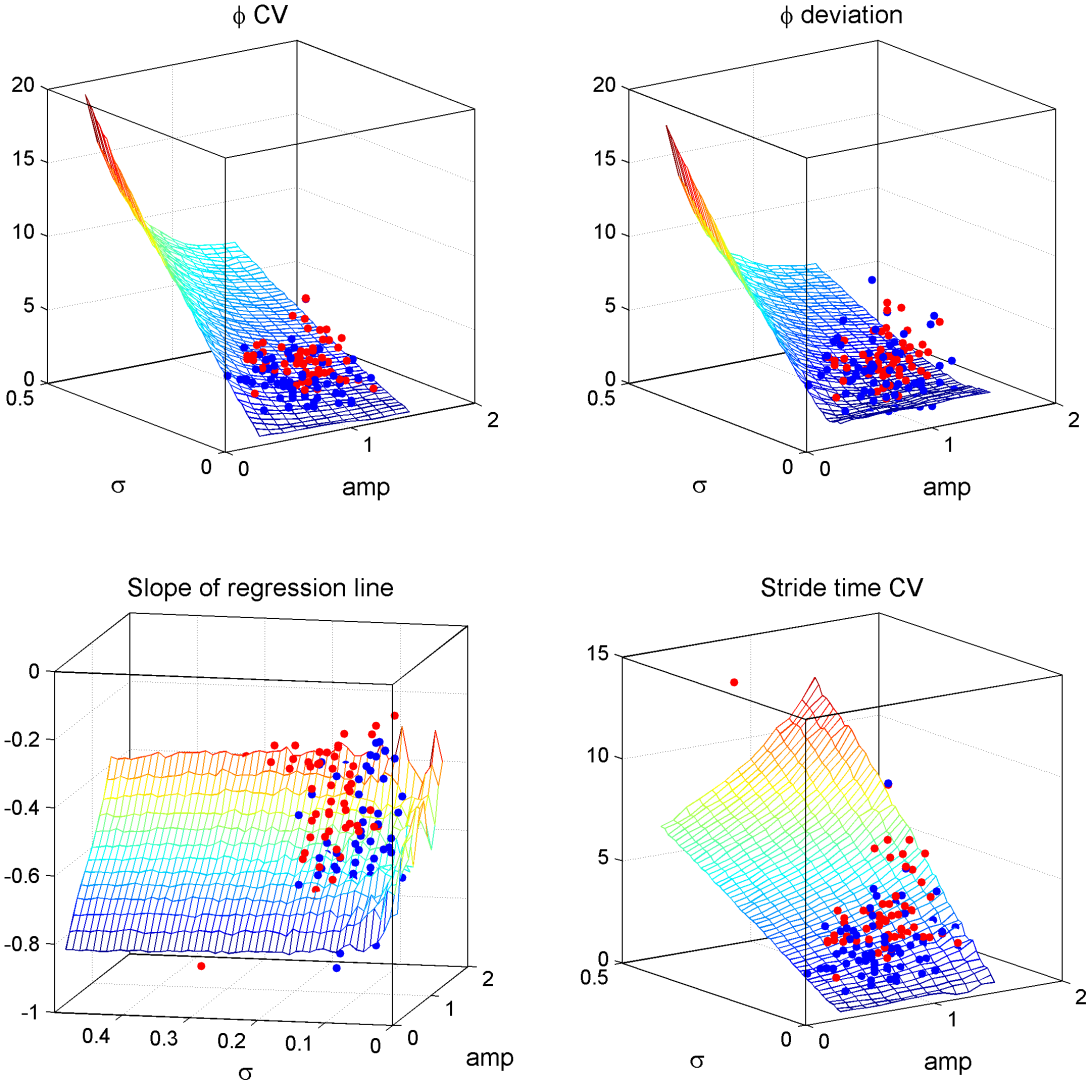
Parameters quantified by the model of coupled phase-oscillators. Mesh figures show how each *φCV*, *φ_deviation*, the slope of the regression line between the relative step phase and the phase change, and *stride time CV* varying according to the strength of the phase reset (*amp*) and the magnitude of noise in the phase reset (*σ*). Strong phase reset (large values of *amp*) decreased *φCV* and *φ_deviation*, and increased *stride time CV*. All three of these parameters increased as noise (*σ*) increased. The slope of regression line between the relative step phase and the phase change decreased with increases in the strength of phase reset. The model mimicking the gait patterns in FOG−P (red dots) showed stronger (larger *amp*) and noisier (larger *σ*) phase reset than the model mimicking the gait patterns in PD−FOG (blue dots). The mesh figures show results from one typical set of model parameters (*ω_1_* = *ω_2_* = 1/2π, *α* = *β* = 0.05), therefore the red and blue dots are not consistent with the values shown by the mesh figures. However, the results obtained from the other set of model parameters also exhibited the same tendency.

In the model of two coupled phase-oscillators, the slope in the regression analysis of Δ*φ_i_* and *φ_i_* was correlated with the strength of the phase reset (Figure 4), and the small negative slope in FOG–P indicated a forceful phase reset. The model that mimicked the results during straight walking showed that the strength and the noise of the phase reset tended to be larger in FOG–P than in PD–FOG, and that the forceful and noisy phase resetting in FOG–P could cause high *stride time CV* (Figure 4). In contrast, *φCV* decreased as the magnitude of phase resetting increased, although it was increased by the noisy phase resetting (Figure 4). The details of this simulation are described in Appendix S1.

There was a main effect of walking condition (‘Go’ and ‘Back’) on *φ_asymmetry* and Δ*φ_error* (*P*<0.05 and *P*<0.01, respectively; Table 2). There was a significant interaction between group and walking condition in Δ*φ_error* (*P*<0.01; Table 2), and Δ*φ_error* was larger during ‘Back’ than during ‘Go’ in FOG–P (*P*<0.01), but not different between the walking conditions in PD–FOG (*P* = 0.79).

## Discussion

In this study we analyzed the gait of patients with FOG but little to no parkinsonism (FOG–P) and found that although the relative step phase [12], [13], [14] and stride time [18] were slightly more variable than in PD patients without FOG (PD–FOG), these differences were not significant. However, the correction of step phase deviation was more forceful and more noisy in FOG–P than in PD–FOG. Phase asymmetry (*φ_deviation* and *φ_asymmetry*) was similar in the two groups, which is in contrast to previous reports showing greater asymmetry in PD patients with FOG (PD+FOG) than PD–FOG [12], [13], [14], [33].

### Step Phase Variability and Deviation

A study of left-right independent pedaling movements [15] reported that alternating leg movements in PD patients were clustered into four patterns. In cluster 1 the relative phase between the legs was stable at around 180°, in cluster 2 the phase was stable but not at 180°, in cluster 3 the phase quasi-periodically varied gradually from 0° to 360°, and in cluster 4 the phase generally varied irregularly but was sometimes fixed. PD–FOG exhibited leg movements in cluster 1 or 2, and PD+FOG exhibited leg movements in cluster 3 or 4 [15]. This indicates that PD–FOG could keep a relatively stable phase relation between the legs even if the phase deviated from 180°. In contrast, in PD+FOG the left and right legs moved independently at different speeds, indicative of poor interlimb coordination and resulting in large phase variability.

In the current study, PD–FOG showed a tendency for smaller step phase variability than FOG–P with comparable deviation of step phase from 180°. Therefore, the gait pattern in PD–FOG corresponded to cluster 2 identified by Abe et al. [15], whereas the gait pattern in FOG–P was similar to cluster 3 or 4. It would be impossible to walk for a substantial period of time with a gait pattern that corresponded to cluster 3 or 4 because the step phase would reach a critically large value where the legs were no longer alternating but were moving almost in-phase, resulting in a cessation of walking. Therefore, patients who exhibit this pattern will inevitably compensate the deviation during straight walking. A neural mechanism of compensation might keep the deviation of step phase close to 180°, but the mechanism is probably inaccurate and cannot prevent high step phase variability. In contrast, even if patients show a step phase that deviates from 180°, they can continue walking in a straight line if this deviation is constant, as in cluster 2.

Previous gait analyses in PD have indicated that both step phase variability and the deviation of step phase from 180° are associated with FOG [12], [13], [14], [34]. However, in the current study step phase variability and step phase deviation were similar between FOG–P and PD+FOG, although step phase variability had a tendency to differ across the groups. This discrepancy may be due to differences in the clinical features of FOG–P and PD+FOG. One of the differences is the severity of parkinsonism. FOG–P in the current study showed little to no parkinsonism, whereas PD+FOG in previous studies showed severe parkinsonism. Parkinsonism was probably associated with the higher step phase variability and larger deviation of step phase from 180° in PD+FOG reported in previous studies. Another difference between FOG–P and PD+FOG is the degree of asymmetry of clinical features, which can be particularly high in PD. Swing time asymmetry [33] reflects the asymmetry of motor function, and is therefore not likely to be large in non-PD patients who have little asymmetry of symptoms, even if they have FOG. The deviation of step phase from 180° is partly associated with swing time asymmetry [12], and thus can be affected by the asymmetry of symptoms.

### Mechanisms of Interlimb Coordination between the Legs

Human gait requires regulation of the step phase between the legs. For example, the step phase should be kept at around 180° during straight walking, but needs to be shifted step-by-step during turning. When the step phase is disturbed unexpectedly such as during stumbling, it is reset to a steady state to prevent falling [28], [29]. The phase reset can be controlled by various components of the central nervous system including a central pattern generator (CPG) in the spinal cord [35] and the locomotor center in the brainstem [36]. CPGs regulate alternating left-right leg movements using reciprocal inhibition [36] and phase resetting [28], [29]. Pedaling leg movement patterns were reproduced by a half-center model of a CPG [37], [38], indicating that interlimb coordination can be regulated by this pathway.

To better understand the mechanisms of interlimb coordination during straight walking we constructed a model of coupled phase-oscillators that used a phase resetting mechanism (Appendix S1). This model reproduced sequences of gait parameters that were similar to those recorded in patients, and verified that the slope of the relation between step phase change (Δ*φ_i_*) and step phase (*φ_i_*) reflected the strength of the phase reset. The slope of the relation between step phase change and step phase was smaller in FOG–P than PD–FOG and the noise in the step phase correction was larger during walking, indicating that the phase reset was more forceful but noisy. Moreover, the model indicated that the noisy phase reset in FOG–P increased variability in step phase and stride time, and that a forceful phase reset could reduce step phase variability but increase stride time variability. A forceful phase reset could stabilize the step phase, but changed the stride time, increasing stride time variability. These results verify that interlimb coordination can influence regulation of the gait cycle.

It is still unknown whether forceful or noisy step phase correction was a primary factor in FOG–P gait. That is, it is unknown whether patients tried to execute a forceful step phase correction because the step phase correction was noisy, or whether the noise was high because of a pathologically impetuous phase correction. However, we believe that noisy interlimb coordination was the primary change, and that the phase correction was forceful in an attempt to reduce the influence of the noise. This is supported by the observation that the noise was larger during the ‘Back’ portion of the walk than during the ‘Go’ portion in FOG–P, despite no difference in the strength of the phase correction between the walking conditions. A turning movement requires asymmetric left-right movement, and thus more precise interlimb coordination than straight walking. Large step phase variability due to noisy interlimb coordination before a turn would interfere with a quick regulation of the step phase for a turn, which makes a turn difficult. During the ‘Go’ portion of the walk patients should tightly regulate the step phase to diminish the influence of noise and enable them to perform the upcoming turn, whereas during the ‘Back’ portion of the walk they could walk and complete the task with a variable step phase. In addition, FOG–P had a tendency for larger step phase variability than PD–FOG. If the forceful phase correction was the primary factor in FOG–P gait, it should have reduced step phase variability according to the model of coupled phase-oscillators and thus step phase variability should have been small in FOG–P.

### Impaired Regulation of Gait Cycle

Hausdorff et al. [18] showed that stride time was more variable in PD+FOG than in PD–FOG. In the current study, FOG–P had a tendency to be larger stride time variability than PD–FOG. *Stride time CV* was used to quantify stride time variability, but this measure can be affected by walking speed. Stride time typically increases as walking speed decreases. A PD–FOG patient in this study (PD 7; Table S2) walked slowly and had a long stride time at the start of the task, but increased walking speed and decreased stride time gradually throughout the walking task. A change in walking speed throughout the task can be associated with a gradual change in stride time, and may influence *stride time CV*, even if gait cycle regulation is intact.

Cadence was higher during turning than during straight walking in PD+FOG, but not in PD–FOG and healthy subjects [19]. Step length becomes shorter when cadence exceeds a certain threshold, and this threshold is lower in PD patients than in age-matched control subjects [39]. Therefore, step length tends to decrease prominently once cadence increases during turning. In addition, a gradual reduction of step length is associated with FOG [40], [41], [42]. These results indicate that gait festination (increased cadence and decreased step length) is associated with FOG [43]. Same phenomena as gait festination were reported during repetitive finger movements [44], [45] and during an orofacial diadochokinetic task [46]. Impaired regulation of rhythmicity during periodic repetitive movements may hasten leg movements during turning, resulting in gait festination and FOG [47].

A reduction in step length was reported to exacerbate gait asymmetry and interlimb incoordination and be a primary cause of FOG [48]. However, patients in these studies were forced to walk with unnaturally short steps, and it is therefore likely that their gait was different from a usual short-stepped gait [49]. Bhatt et al. [50] reported that the reduction in step length during turning was similar in PD+FOG, PD–FOG and healthy subjects, and that it was increased stride time variability that was associated with FOG.

### Mechanisms Underlying FOG

One possible mechanism of FOG involves dysfunction of CPGs or the brainstem locomotor center that leads to inaccurate correction or poor detection sensitivity of deviation of step phase from 180° and thus leads to noisy interlimb coordination. The large amount of noise increases activation of these locomotor centers; this abnormal activation leads to impetuous phase corrections in an attempt to reduce the impact of the noise, and these impetuous phase corrections increase stride time variability. In addition, patients might choose to reduce step length and stride time to diminish the detrimental consequences of the noise [51]. These responses cause hastened leg movements and gait festination, leading to FOG, especially in conditions that require precise interlimb coordination, such as turning. FOG–P and PD+FOG exhibited different clinical features and gait parameters such as step phase variability and stride time variability, and the mechanisms of FOG may differ between FOG–P and PD+FOG. Future studies should investigate interlimb coordination during turning, and should investigate the hastened movements that occur in FOG patients during leg repetitive movements such as foot tapping.

## Conclusions

FOG patients with little to no parkinsonism showed forceful and noisy interlimb coordination during straight walking, which was probably due to dysfunction of the brainstem locomotor center or CPGs. This interlimb coordination was associated with high stride time variability, which may have caused gait festination and FOG during turning. These findings were supported by a model of coupled phase-oscillators that used the phase resetting mechanism. The mechanisms underlying FOG may differ between FOG patients with little to no parkinsonism and PD patients with FOG. Interlimb coordination during turning and leg rhythmicity during repetitive movements should be investigated in FOG patients to more precisely understand the mechanisms of FOG.

## Supporting Information

Figure S1 **A model of two coupled phase-oscillators.** Two oscillators rotated at constant angular velocities (*ω*_1_, *ω*_2_). When the phase of one oscillator reached 2π rad ( = 0) this was interpreted as corresponding to a foot contact event during walking and the phase of the other oscillator was reset close to π rad. This phase reset creates alternating oscillations of two oscillators. *θ* ^(2)^ is the phase of oscillator-2 before phase reset performed when the foot contact event occurred in oscillator-1, i.e., when *θ* ^(1)^ = 2π, Δ*θ* ^(2)^ is the magnitude of the phase reset, and *θ* ^(2)^(*t_n_*_+_) is the phase of oscillator-2 after the phase reset. *amp_1→2_* defines the strength of the phase reset, and *β* is the deviation of the equilibrium phase from π. For example, if the phase of oscillator-2 already passed the equilibrium point (π+*β*) at the time of the foot contact event in oscillator-1 (*θ* ^(1)^ = 2π), sin (*θ* ^(2)^–*β*) is a negative value and the phase of oscillator-2 is returned close to the equilibrium phase.
(TIFF)

Figure S2 **Representative sequences of parameters obtained from the model of coupled phase-oscillators.** The cycle time of each oscillator (upper), the relative phase between the two oscillators (middle), and the phases of each oscillator at the onset of phase reset (lower) when the model parameters were set to reproduce the gait patterns observed in the ‘Go’ portion of the walking task performed by the PD–FOG patient (left) and FOG–P patient (right) shown in Figure 2. Cycle time and relative phase correspond to stride time and relative step phase in gait analysis. These sequences showed a tendency similar to the patients’ results. The model parameters required to reproduce the gait pattern of the PD–FOG patient were *ω_1_* = 1/2π, *ω_2_* = 0.95/2π, *amp_1→2_* = 0.6, *amp_2→1_* = 0.7, *α* = *β* = –0.05, and *σ* = 0.125. The model parameters required to reproduce the gait pattern of the FOG–P patient were *ω_1_* = 1/2π, *ω_2_* = 0.95/2π, *amp_1→2_* = 1.5, *amp_2→1_* = 0.9, *α* = *β* = 0, and *σ* = 0.175.
(TIF)

Table S1 **Clinical features of each patient.**
(XLSX)

Table S2 **Gait parameters of each patient during straight walking.**
(XLSX)

Appendix S1 **A model of coupled phase-oscillators interacting through a phase resetting mechanism.**
(DOCX)
