# Supplementary figures and images for: Noisy Interlimb Coordination Can Be a Main Cause of Freezing of Gait in Patients with Little to No Parkinsonism
Source: PLoS One. 2013 Dec 31;8(12):e84423. doi: 10.1371/journal.pone.0084423 (PMC3877290; doi:10.1371/journal.pone.0084423)

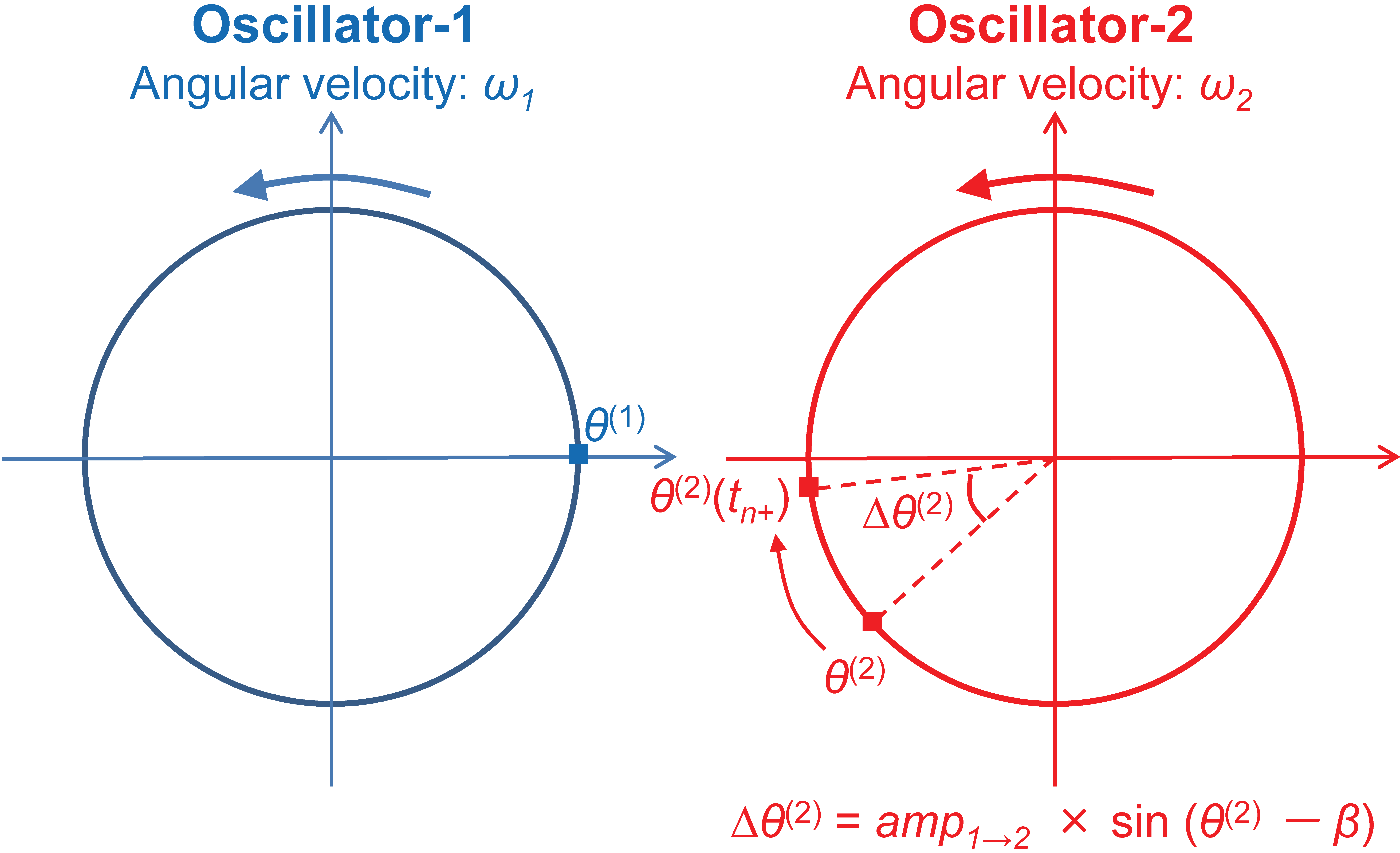

Supplement: Figure S1 — A model of two coupled phase-oscillators. Two oscillators rotated at constant angular velocities (ω 1, ω 2). When the phase of one oscillator reached 2π rad ( = 0) this was interpreted as corresponding to a foot contact event during walking and the phase of the other oscillator was reset close to π rad. This phase reset creates alternating oscillations of two oscillators. θ (2) is the phase of oscillator-2 before phase reset performed when the foot contact event occurred in oscillator-1, i.e., when θ (1) = 2π, Δθ (2) is the magnitude of the phase reset, and θ (2)(tn +) is the phase of oscillator-2 after the phase reset. amp1→2 defines the strength of the phase reset, and β is the deviation of the equilibrium phase from π. For example, if the phase of oscillator-2 already passed the equilibrium point (π+β) at the time of the foot contact event in oscillator-1 (θ (1) = 2π), sin (θ (2)–β) is a negative value and the phase of oscillator-2 is returned close to the equilibrium phase. (TIFF) [file pone.0084423.s001.tiff]

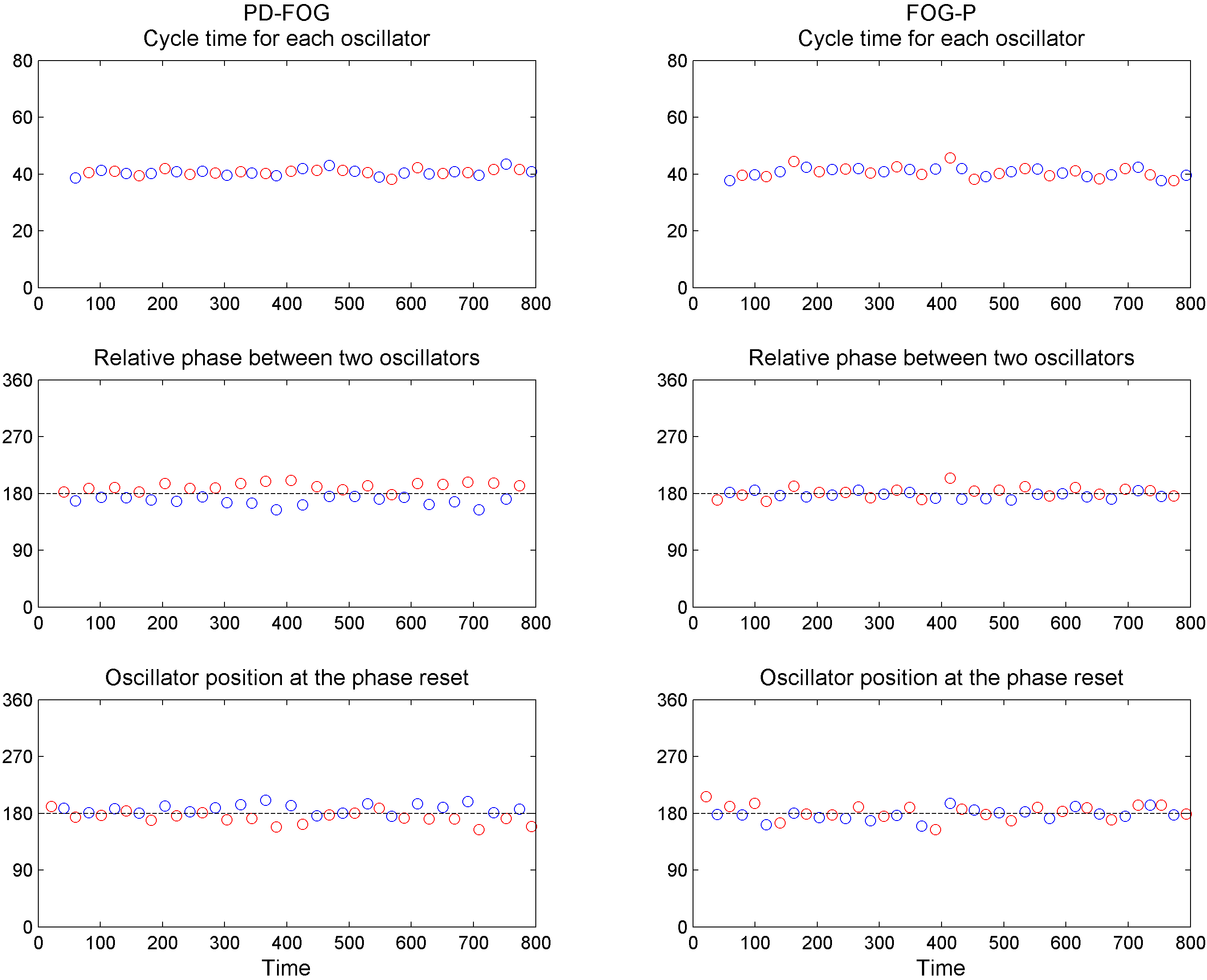

Supplement: Figure S2 — Representative sequences of parameters obtained from the model of coupled phase-oscillators. The cycle time of each oscillator (upper), the relative phase between the two oscillators (middle), and the phases of each oscillator at the onset of phase reset (lower) when the model parameters were set to reproduce the gait patterns observed in the ‘Go’ portion of the walking task performed by the PD–FOG patient (left) and FOG–P patient (right) shown in Figure 2. Cycle time and relative phase correspond to stride time and relative step phase in gait analysis. These sequences showed a tendency similar to the patients’ results. The model parameters required to reproduce the gait pattern of the PD–FOG patient were ω1 = 1/2π, ω2 = 0.95/2π, amp1→2 = 0.6, amp2→1 = 0.7, α = β = –0.05, and σ = 0.125. The model parameters required to reproduce the gait pattern of the FOG–P patient were ω1 = 1/2π, ω2 = 0.95/2π, amp1→2 = 1.5, amp2→1 = 0.9, α = β = 0, and σ = 0.175. (TIF) [file pone.0084423.s002.tif]
