# Supplementary material for: Noisy Interlimb Coordination Can Be a Main Cause of Freezing of Gait in Patients with Little to No Parkinsonism
Source: PLoS One. 2013 Dec 31;8(12):e84423. doi: 10.1371/journal.pone.0084423 (PMC3877290; doi:10.1371/journal.pone.0084423)
Supplement: Appendix S1 — A model of coupled phase-oscillators interacting through a phase resetting mechanism. (DOCX) [file pone.0084423.s005.docx]

**Appendix**

**Phase resetting curves and a coupled phase-oscillators model**

A model of two coupled phase-oscillators was developed to shed light on the mechanisms of regulation of step phase and stride time during straight walking and to provide a theoretical basis of the gait analysis performed in the main text. In this model, the state of each oscillator rotated around a unit circle at a constant angular velocity (*ω*_1_, *ω*_2_; Figure S1). The states of oscillator-1 and oscillator-2 at time *t* were described by their oscillation phases, *θ*^(1)^(*t*) and *θ*^(2)^(*t*), respectively, corresponding to the gait phases of the left and right legs during walking. If the two oscillators oscillated independently with no interaction, they could not exhibit stable alternating oscillations.

We considered an event to occur when the phase of each oscillator reached 0 rad (= 2π). This event corresponds to a foot contact during walking. The two oscillators interacted impulsively with each other for a sequence of foot contact events. A foot contact event in oscillator-1 ($\theta^{(1)}=0$) was transmitted to oscillator-2 and instantaneously modified the phase of oscillator-2 (*θ*^(2)^) by the amount of *∆θ*^(2)^, and in the same way an event in oscillator-2 ($\theta^{(2)}=0$) modified the phase of oscillator-1 (*θ*^(1)^) by the amount of *∆θ*^(1)^. The amount of phase modification, referred to as phase reset [28], [29], is usually dependent on the phase at the time of reset: $\Delta\theta^{(1)}=\Delta\theta^{(1)}(\theta^{(1)})$ and $\Delta\theta^{(2)}=\Delta\theta^{(2)}(\theta^{(2)})$. Graphs of $\Delta\theta^{(1)}=\Delta\theta^{(1)}(\theta^{(1)})$ as a function of *θ*^(1)^ and $\Delta\theta^{(2)}=\Delta\theta^{(2)}(\theta^{(2)})$ as a function of *θ*^(2)^ are referred to as phase resetting curves (PRCs). Typical PRCs that cause alternating oscillations in the model can be formulated as follows.

$$\Delta\theta^{(1)}(\theta^{(1)})=amp_{2\to1}\times\sin(\theta^{(1)}+\alpha)$$

$$\Delta\theta^{(2)}(\theta^{(2)})=amp_{1\to2}\times\sin(\theta^{(2)}-\beta)$$

*θ*^(1)^ is the phase of oscillator-1 immediately before oscillator-1 is reset by the event $\theta^{(2)}=0$, and *θ*^(2)^ is the phase of oscillator-2 immediately before oscillator-2 is reset by the event $\theta^{(1)}=0$. The parameters *amp_2→1_* and *amp_1→2_* define the magnitude of the interaction from oscillator-2 to oscillator-1 and from oscillator-1 to oscillator-2 respectively through the phase reset. The parameters *α* and *β*, which may be close to 0, determine the equilibrium phase, where the phase resetting is zero. Dynamics of the coupled phase-oscillators can be described by a sequence of the phases of two oscillators immediately before each oscillator is reset.

We considered the phase of oscillator-1, *θ_n_*^(1)^, immediately before oscillator-1 was reset by the *n*-th foot contact event in oscillator-2, and the phase of oscillator-2, *θ_m_*^(2)^, immediately before oscillator-2 was reset by the *m*-th foot contact event in oscillator-1. The subscripts *n* and *m* are used only for the phases when the *n*-th or the *m*-th foot contact event occurs in the contra-lateral oscillator and the resultant phase reset takes place. After walking begins, the first foot contact event in oscillator-1 occurs at time $t=t_{1}$, and oscillator-2 located at *θ*_1_^(2)^ is reset to $\theta^{(2)}(t_{1+})=\theta_{1}^{(2)}+amp_{1\to2}\times\sin(\theta_{1}^{(2)}-\beta)$, where *t*_1+_ with the subscript ‘’+’’ indicates the time at *t*_1_ but immediately after the phase reset. At this moment, oscillator-1 is still located at 0 rad ($\theta^{(1)}(t_{1+})=0$). The second foot contact event occurs in oscillator-2 at $t=t_{2}$ when *θ*^(2)^(*t*_2_) reaches 2π. The phase of oscillator-1 at $t=t_{2}$ can be determined based on the time interval from *t*_1_ to *t*_2_. The co-phase of oscillator-2, i.e. the remaining phase for oscillator-2 to reach 2π from *θ*^(2)^(*t*_1+_) immediately after the phase reset, is $2\pi-\theta^{(2)}(t_{1+})$. Thus, the time necessary for oscillator-2 to reach 2π is$\{2\pi-\theta^{(2)}(t_{1+})\}/\omega_{2}$, during which the phase of oscillator-1 evolves from 0 to $\{2\pi-\theta^{(2)}(t_{1+})\}\times\omega_{1}/\omega_{2}$. Therefore, oscillator-1 is reset by oscillator-2 at time *t*_2_ when oscillator-1 is located at $\theta_{2}^{(1)}=\theta^{(1)}(t_{2})$, which is determined as follows.

$$\begin{matrix} \theta_{2}^{(1)}=\{2\pi-\theta^{(2)}(t_{1+})\}\times\omega_{1}/\omega_{2} (\mathrm{mod} 2\pi) \\ =\{2\pi-\{\theta_{1}^{(2)}+amp_{1\to2}\times\sin(\theta_{1}^{(2)}-\beta)\}\}\times\omega_{1}/\omega_{2} (\mathrm{mod} 2\pi) \end{matrix}$$

In a similar way, the third foot contact event occurs in oscillator-1 at $t=t_{3}$ when *θ*^(1)^(*t*_3_) reaches 2π. The co-phase of *θ*^(1)^(*t*_2+_) to reach 2π is $\{2\pi-\theta^{(1)}(t_{2+})\}/\omega_{1}$, during which the phase of oscillator-2 evolves from 0 to $\{2\pi-\theta^{(1)}(t_{2+})\}\times\omega_{2}/\omega_{1}$. Thus, oscillator-2 is reset by oscillator-1 at time *t*_3_ when oscillator-2 is located at$\theta_{3}^{(2)}=\theta^{(2)}(t_{3})$, which is determined as follows.

$$\begin{matrix} \theta_{3}^{(2)}=\{2\pi-\theta^{(1)}(t_{2+})\}\times\omega_{2}/\omega_{1} (\mathrm{mod} 2\pi) \\ =\{2\pi-\{\theta_{2}^{(1)}+amp_{2\to1}\times\sin(\theta_{2}^{(1)}+\alpha)\}\}\times\omega_{2}/\omega_{1} (\mathrm{mod} 2\pi) \end{matrix}$$

Deductive calculation shows that

$$\theta_{n+1}^{(1)}=\{2\pi-\theta_{n}^{(2)}-amp_{1\to2}\times\sin(\theta_{n}^{(2)}-\beta)\}\times\omega_{1}/\omega_{2}+\varepsilon_{n}^{(2)} (\mathrm{mod} 2\pi)$$

$$\theta_{n+2}^{(2)}=\{2\pi-\theta_{n+1}^{(1)}-amp_{2\to1}\times\sin(\theta_{n+1}^{(1)}+\alpha)\}\times\omega_{2}/\omega_{1}+\varepsilon_{n+1}^{(1)} (\mathrm{mod} 2\pi)$$

where *ε_n_*^(2)^ and *ε_n_*_+1_^(1)^ are Gaussian white noise in the phase reset. The noise intensity depends on the standard deviations (σ) of *ε*^(1)^ and *ε*^(2)^.

**Regression analysis of relative step phase**

In the main text, linear regression analyses were performed between step phase change (*∆φ_i_*) and step phase (φ_i_). Here we consider the dynamics of the coupled phase-oscillators model in terms of the relative step phase, and clarify how the regression analyses of the relative step phase were related to the PRCs in terms of the phase *θ_n_*^(1)^ and *θ_m_*^(2)^ in the model.

We considered occurrence of the (*n*+1)-th foot contact event in oscillator-1 at $t=t_{n+1}$. At this moment, oscillator-1 and oscillator-2 are located at the phases *θ*^(1)^(*t_n_*_+1_) (= 0) and *θ_n_*_+1_^(2)^, respectively. *t_n_* and *t_n_*_+2_ denote the time instants of the preceding and subsequent foot contact event in oscillator-2. The phase of oscillator-2 evolves from $\theta^{(2)}(t_{n})=0$ to *θ_n_*_+1_^(2)^ in the time interval of [*t_n_*, *t_n_*_+1_] during which no phase reset occurs in oscillator-2. Thus, the time interval of [*t_n_*, *t_n_*_+1_] is $\theta_{n+1}^{(2)}/\omega_{2}$. The phase of oscillator-2 evolves from *θ_n_*_+1_^(2)^ to $\theta^{(2)}(t_{n+2})=2\pi$ in the time interval of [*t_n_*_+1_, *t_n_*_+2_] during which (at $t=t_{(n+1)+}$) the phase of oscillator-2 is reset by the amount of *∆θ*^(2)^(*θ_n_*_+1_^(2)^). Thus, the precise amount of phase evolution (co-phase) necessary for oscillator-2 to reach 2π is $2\pi-\{\theta_{n+1}^{(2)}+\Delta\theta^{(2)}(\theta_{n+1}^{(2)})\}$, and the time interval of [*t_n_*_+1_, *t_n_*_+2_] is $2\pi-\{\theta_{n+1}^{(2)}+\Delta\theta^{(2)}(\theta_{n+1}^{(2)})\}/\omega_{2}$. Therefore, the relative step phase of the (*n*+1)-th foot contact event in oscillator-1 (*φ_n_*_+1_^(1)^) was represented as follows.

$$\begin{matrix} \phi_{n+1}^{(1)}=(t_{n+1}-t_{n})/(t_{n+2}-t_{n}) \\ =(t_{n+1}-t_{n})/\{(t_{n+1}-t_{n})+(t_{n+2}-t_{n+1})\} \\ =\theta_{n+1}^{(2)}/\{\theta_{n+1}^{(2)}+\{2\pi-\{\theta_{n+1}^{(2)}+\Delta\theta^{(2)}(\theta_{n+1}^{(2)})\}\}\} \\ =\theta_{n+1}^{(2)}/\{2\pi-\Delta\theta^{(2)}(\theta_{n+1}^{(2)})\} \end{matrix}$$

However, when we consider the regulation of relative step phase during walking, there is no information about the phase of oscillator-2 (*θ_n_*_+1_^(2)^) and the time of the (*n*+2)-th foot contact event in oscillator-2 (*t_n_*_+2_) at the time of the (*n*+1)-th foot contact event in oscillator-1 (*t_n_*_+1_). Therefore, the time interval of [*t_n_*, *t_n_*_+2_] was approximated by the original time for one cycle of oscillator-2, $T_{2}=2\pi/\omega_{2}$, by which the relative step phase of the (*n*+1)-th foot contact event in oscillator-1 was represented as follows.

$$\begin{matrix} \phi_{n+1}^{(1)}=(t_{n+1}-t_{n})/T_{2} \\ =(\theta_{n+1}^{(2)}/\omega_{2})/(2\pi/\omega_{2}) \\ =\theta_{n+1}^{(2)}/2\pi\end{matrix}$$

In a similar way, the relative step phase of the (*n*+2)-th foot contact event in oscillator-2 was represented using the original time for one cycle of oscillator-1, $T_{1}=2\pi/\omega_{1}$, as follows.

$$\begin{matrix} \phi_{n+2}^{(2)}=(t_{n+2}-t_{n+1})/T_{1} \\ =\{2\pi-\{\theta_{n+1}^{(2)}+\Delta\theta^{(2)}(\theta_{n+1}^{(2)})\}\}/\omega_{2}/(2\pi/\omega_{1}) \\ =\{2\pi-\{\theta_{n+1}^{(2)}+\Delta\theta^{(2)}(\theta_{n+1}^{(2)})\}\}\times\omega_{1}/\omega_{2}/2\pi\end{matrix}$$

Therefore, the phase change (*∆φ_n_*_+1_) from the step phase for the (*n*+1)-th foot contact event in oscillator-1 (*φ_n_*_+1_^(1)^) to the phase for the (*n*+2)-th foot contact event in oscillator-2 (*φ_n_*_+2_^(2)^) was approximated as follows.

$$\begin{matrix} \Delta\phi_{n+1}=\phi_{n+2}^{(2)}-\phi_{n+1}^{(1)} \\ =\{2\pi-\{\theta_{n+1}^{(2)}+\Delta\theta^{(2)}(\theta_{n+1}^{(2)})\}\}\times\omega_{1}/\omega_{2}/2\pi-\theta_{n+1}^{(2)}/2\pi\\ =\{\{2\pi-\{\theta_{n+1}^{(2)}+amp_{1\to2}\times\sin(\theta_{n+1}^{(2)}-\beta)\}\}\times\omega_{1}/\omega_{2}-\theta_{n+1}^{(2)}\}/2\pi\end{matrix}$$

The slope of the regression between step phase and phase change was calculated by differentiating this equation.

$$d(\Delta\phi_{n+1})/d(\theta_{n+1}^{(2)})=\{-\omega_{1}/\omega_{2}\times\{1+amp_{1\to2}\times\cos(\theta_{n+1}^{(2)}-\beta)\}-1\}/2\pi$$

*θ_n_*_+1_^(2)^ varied around 180° and *β* was close to 0, therefore cos (*θ_n_*_+1_^(2)^ – *β*) was approximated as – 1.

$$\begin{matrix} d(\Delta\phi_{n+1})/d(\theta_{n+1}^{(2)})\approx\{-\omega_{1}/\omega_{2}\times(1-amp_{1\to2})-1\}/2\pi\\ \approx\{\omega_{1}/\omega_{2}\times amp_{1\to2}-(\omega_{1}/\omega_{2}+1)\}/2\pi\end{matrix}$$

*ω*_1_ and *ω*_2_ are positive values, therefore the slope of regression line was positively correlated with the strength of phase reset (*amp_1→2_*). The slope was generally smaller than zero, therefore a small negative slope of the regression line indicated a large strength of phase reset (large value of *amp_1→2_*).

**Typical dynamics of the model mimicking PD−FOG and FOG−P behaviors**

Figure S2 shows representative sequences of the phase of each oscillator at the onset of phase reset (*θ_n_*^(1)^, *θ_m_*^(2)^), the relative phase (*φ*^(1)^, *φ*^(2)^) and the time for one cycle of each oscillator (cycle time). These sequences showed similar tendency to patients’ results during the ‘Go’ portion of the walking task (Figure 2).

Gait parameter equivalents (*φCV*, *φ_deviation*, and *stride time CV*) were calculated from simulated sequences. Each parameter changed as the strength (*amp*) and noise (*σ*) of the phase reset changed (Figure 4). An increase in the strength of phase reset decreased *φCV* and *φ_deviation*, but increased *stride time CV* because the phase reset changed cycle time (stride time). In contrast, noise of the phase reset increased *φCV*, *φ_deviation* and *cycle time CV*. The slope of the regression between step phase (*φ_i_*) and phase change (*∆φ_i_*) decreased as the strength of phase reset increased, as considered theoretically above, but slightly increased as noise of the phase reset increased. The asymmetry of the intercept (*φ_asymmetry* in the main text) depended on the deviation of the equilibrium phase from π (*α*, *β*) and the difference between the angular velocities of the two oscillators (*ω*_1_, *ω*_2_). Moreover, the asymmetry was influenced by the strength and noise of phase reset, but the influence varied depending on the parameters of the model, and did not show a constant tendency.

We examined dynamics of the model with various parameter values, varying the angular velocity of each oscillator (*ω*_1_, *ω*_2_), the strength of phase reset (*amp_1→2_*, *amp_2→1_*), the deviation of the equilibrium phase from π (*α*, *β*), and the noise intensity in the phase reset (*σ*). We confirmed that the gait pattern of every patient could be reproduced by the model for a set of patient-dependent parameter values. Models that mimicked the gait patterns in FOG–P had more forceful and more noisy phase reset than models that mimicked the gait patterns in PD–FOG (Figure 4).

We verified that the regression between step phase (*φ_i_*) and phase change (*∆φ_i_*) is a reasonable tool for evaluating interlimb coordination. The slope of the regression line was smaller in FOG–P than in PD–FOG and the noise of phase reset was larger, indicating the presence of an forceful but noisy phase reset in FOG–P. The large noise caused high step phase variability in FOG–P, and the forceful phase reset caused high stride time variability, probably making patients susceptible to gait festination and FOG.

There are two limitations of this model. One is the lack of consideration of impaired rhythmicity that appears during unilateral limb movements such as foot tapping. Each oscillator in the model rotates at a constant angular velocity, but legs do not always move at a constant velocity during walking, and interlimb coordination is affected by the impaired rhythmicity. Another limitation is the lack of consideration of sensory feedback. The phase reset in the model is performed at the moment of the foot contact event in each oscillator, but the time delay and the sensitivity of sensory feedback should be considered, and are possibly different between the two legs. The simple model was sufficient to understand interlimb coordination in this study, but a more complex model that includes these factors should be investigated in future studies, and a model of gait during turning should be also constructed.
